# Optimizing Diabetic Macular Edema Treatment: A Meta-Analysis of Subthreshold Micropulse Laser and Anti-Vascular Endothelial Growth Factor Combination Therapy

**DOI:** 10.3390/jcm13164782

**Published:** 2024-08-14

**Authors:** Ching-Chih Ma, Po-Huang Chen, Yun-Hsiu Hsieh

**Affiliations:** 1 Department of Ophthalmology, Tri-Service General Hospital, National Defense Medical Center, Taipei 114, Taiwan; sqries7231@gmail.com; 2 Department of Internal Medicine, Tri-Service General Hospital, National Defense Medical Center, Taipei 114, Taiwan

**Keywords:** diabetic macular edema, subthreshold micropulse laser, anti-VEGF

## Abstract

**Background:** Diabetic macular edema (DME) is the primary cause of visual impairment in individuals with diabetes. Anti-vascular endothelial growth factor (VEGF) is the current first-line treatment for DME owing to its effectiveness. However, frequent anti-VEGF injections may be inconvenient for patients. Therefore, this study aimed to investigate whether the addition of subthreshold micropulse laser (SML) to anti-VEGF therapy could reduce the requirement for anti-VEGF injections while maintaining the treatment efficacy for DME. **Methods:** Clinical trials retrieved from the databases of PubMed, EMBASE, and the Cochrane Library were evaluated to determine the effectiveness of combination treatment with SML and anti-VEGF medication compared with that of anti-VEGF treatment alone. The primary outcome measures were the changes in CMT, best-corrected visual acuity (BCVA), and the total number of intravitreal injections (IVIs). **Results:** The IVI + SML group revealed a substantial increase in the logarithm of the minimum angle of the resolution BCVA and a reduction in CMT at the 12-month follow-up (BCVA: random-effects; mean difference [MD], −0.05; 95% confidence interval [CI]: −0.10 to −0.01; *p*-value = 0.28, and CMT: random-effects; MD, −18.27; 95% confidence interval, −27.36 to −9.18; *p*-value = 0.20). The number of required IVIs in the IVI + SML group was lower than that in the IVI only group (random-effects; MD, −2.22; 95% CI: −3.13 to −1.31; *p*-value < 0.01). **Conclusions:** Combining SML therapy with anti-VEGF injections may reduce the total number of injections required, improve VA, and reduce CMT at the 12-month follow-up. Although the included studies used different SML regimens and anti-VEGF agents, this review indicates that the application of additional SML therapy results in positive clinical outcomes.

## 1. Introduction

Diabetic macular edema (DME), which can develop at any stage of diabetic retinopathy, is a common cause of impaired vision in patients with diabetes. DME is prevalent in 14.3% and 5.6% of patients with type 1 diabetes and type 2 diabetes, respectively [1,2]. It is also associated with a longer duration of diabetes, poor glycated hemoglobin (HbA1c) control, hypertension, and dyslipidemia. The pathogenesis of DME is a multifactorial process triggered by hyperglycemia. Vascular endothelial growth factor (VEGF) is a potent vasopermeability factor that results in the breakdown of the blood–retinal barrier and the subsequent development of DME [2,3]. The treatments for DME include laser photocoagulation, intravitreal injection (IVI) of steroids or anti-VEGF, and vitrectomy [4]. The EDTRS trial reported that conventional macular laser photocoagulation reduced the risk of vision loss by 50% at the 3-year follow-up visit [5]. However, it can cause visible laser scars, visual field loss, choroidal neovascularization, and subretinal fibrosis [6,7,8,9]. The treatment trend for centers involving DME appears to be shifting from a laser ablation approach toward pharmacotherapy, especially the IVI of anti-VEGF agents [4]. Several clinical trials have demonstrated the efficacy of anti-VEGF therapy in improving vision in DME patients [10,11,12,13,14], leading to its recommendation as the first-line therapy in the guidelines of the AAO, EURETINA, and ASRS [15,16,17]. Expert consensus in Taiwan also supports anti-VEGF as the primary treatment for centers involving DME, emphasizing that early intensive therapy, with at least 3 monthly injections, is crucial for significant improvements in visual acuity and anatomical outcomes. [18]. Although IVI of anti-VEGF agents can rapidly improve visual acuity [19], it requires regular injection, which increases the risk of infection, retinal detachment, and cataract formation [20,21]. Furthermore, frequent IVI of anti-VEGF agents is costly and inconvenient.

The use of subthreshold micropulse lasers (SMLs) has been proposed due to the adverse events associated with conventional macular laser photocoagulation. Compared with conventional macular lasers, SMLs have longer wavelengths and enable micropulse delivery. Furthermore, there is no visible laser scarring or detectable retinal damage on multimodal imaging [22,23,24,25]. Thus, it is an effective treatment for DME that is superior to conventional macular lasers [26,27,28,29,30,31]. However, SMLs cannot replace pharmacotherapy and can only be used as an adjunctive therapy. SML has been used to treat several retinal disorders, including central serous chorioretinopathy, DME, proliferative diabetic retinopathy, and macular edema secondary to retinal vein occlusion [32]. The main wavelengths for laser treatment in DME are 577 nm yellow light and 532 nm green light, with the former being safer for the macula owing to lower absorption by macular carotenoids, which reduces central retinal damage risk [32,33,34]. While there is no standardized DME treatment protocol, a 577 nm laser with a 200 μm spot diameter, 5% duty cycle, 200 ms duration, and 400 mW power is considered safe and effective for DME with a central macular thickness below 400 μm [35]. Several studies have demonstrated that combination therapy with SML and anti-VEGF drugs may have a positive impact on visual acuity (VA) and central macular thickness (CMT). It may also alleviate the treatment burden associated with anti-VEGF injections [36,37,38,39,40,41,42,43,44,45]. Therefore, this study aimed to investigate the potential of combination therapy with SML and anti-VEGF agents to reduce the number of anti-VEGF injections required while maintaining treatment efficacy for DME. The treatment outcomes associated with combination therapy were evaluated, and the feasibility of such a combination as an alternative to traditional treatments was analyzed.

## 2. Materials and Methods

This study was conducted in accordance with the Preferred Reporting Items for Systematic Reviews and Meta-Analyses guidelines for systematic reviews and meta-analyses of randomized controlled trials (RCTs) [46] (Table S1) and the Meta-analysis of Observational Studies in Epidemiology guidelines for observational studies (Table S2) [47]. The protocol used in this systematic review was registered in the Open Science Framework (Charlottesville, VA, USA) (https://osf.io/9h8va (accessed on 22 April 2024)).

### 2.1. Search Strategy

**Figure 1:**
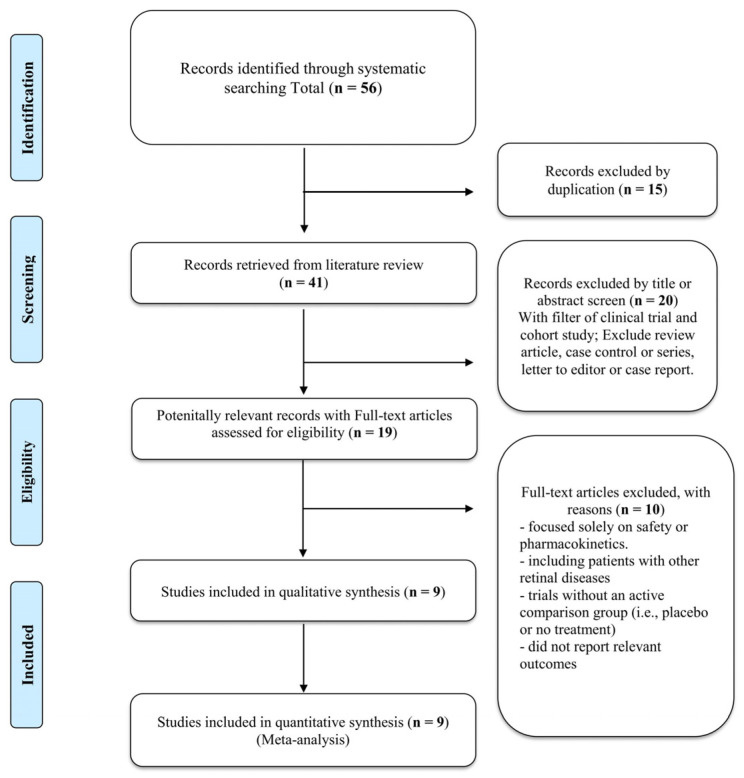
PRISMA flow diagram of the database search and screening processes.

The databases of PubMed, EMBASE, and Cochrane Library were comprehensively searched from the date of database inception to October 2023 (Table S3) using the keywords “diabetic macular edema”, “micropulse laser treatment”, “intravitreal injections”, and “anti-VEGF therapy”. There were no restrictions on language. Figure 1 details the search strategy. In addition to the electronic search, conference abstracts and the references cited in relevant studies were manually searched to identify any potentially relevant studies and reviewed. The search strategy was designed to be as thorough and inclusive as possible to ensure that all relevant studies were included. Studies investigating the potential of combination therapy with SML and anti-VEGF agents to reduce the number of anti-VEGF injections required while maintaining treatment efficacy for DME were eligible for inclusion in this study. The inclusion criteria were as follows: (1) RCTs or observational studies comparing combination therapy with SML and anti-VEGF agents with standard treatments for DME, including monotherapy with anti-VEGF agents or IVIs of steroids or other agents; (2) studies including patients diagnosed with DME, with or without concomitant diabetic retinopathy, regardless of age or sex; and (3) studies reporting outcomes, such as VA, CMT, number of anti-VEGF injections, and adverse events associated with treatment. The exclusion criteria were as follows: (1) studies that did not report outcomes relevant to the meta-analysis, such as studies focusing solely on safety or pharmacokinetics; (2) studies including patients with other retinal diseases, such as age-related macular degeneration or retinal vein occlusion; and (3) case reports, case series, review articles, and editorials that did not meet the criteria for original research studies.

### 2.2. Data Collection

Three independent reviewers (CCM, PHC, and YHH) conducted a comprehensive review of the identified studies based on the selection criteria. Any discrepancies in study selection or data extraction were resolved by reaching a consensus via discussion, and a third-party decision was made if necessary. Information on the following factors was collected: study-related characteristics (including study design, publication year, and country), participant-related characteristics (including mean age, percentage of male participants, population type, preoperative best-corrected visual acuity [BCVA], CMT, HbA1c levels, and pseudophakia status), and treatment-related characteristics (such as the type of anti-VEGF agent used, SML wavelength [nm], postoperative BCVA, CMT, and number of IVIs required).

Dichotomous outcomes were extracted using the number of events and numbers, whereas continuous outcomes were extracted using the number of participants and mean values with standard deviations for outcome measurement in the experimental and comparator groups. A standardized form was used, and the data were independently collected by both reviewers to ensure accuracy and minimize errors during data collection. Discrepancies and uncertainties were resolved by reaching a consensus. The collected data were synthesized and analyzed to provide a comprehensive summary of the available evidence on the effectiveness of combination therapy with SML and anti-VEGF agents for DME.

### 2.3. Quality Assessment

The methodological quality of each study was assessed independently by three reviewers (CCM, YHH, and PHC) using the Newcastle–Ottawa scale for observational studies [48] and the Cochrane Collaboration Risk of Bias tool for RCTs.

### 2.4. Outcome Measurement

The outcomes of interest in this study included the changes in CMT, BCVA, and the total number of IVIs required. CMT was measured using spectral-domain optical coherence tomography at the 3-, 6-, and 12-month follow-up visits. BCVA was measured using the logarithm of the minimum angle of resolution (logMAR) chart at the same follow-up time points. The mean changes in CMT and logMAR BCVA from baseline were calculated for each treatment group at each follow-up visit. The total number of IVIs received by each patient during the study period was also recorded.

### 2.5. Subgroup and Sensitivity Analyses

A subgroup analysis of studies that reported SML wavelengths of 532, 577, and 810 nm was performed to explore the potential impact of different SML wavelengths on treatment outcomes. For different protocol sensitivity analyses, studies that used a treatment protocol that differed significantly from the standard protocols used in most studies were excluded. The remaining studies were then re-analyzed to assess their impact on the overall treatment effect estimates. The results of these analyses are presented as forest plots to facilitate comparisons across subgroups and sensitivity analyses.

### 2.6. Statistical Analysis

Patient data from each included study were pooled and presented as the mean (standard deviation) or number in accordance with the parameter characteristics. Statistical analyses were performed in accordance with the Cochrane Handbook for Systematic Reviews of Interventions [49]. For dichotomous variables, odds ratios with 95% confidence intervals (CIs) were calculated using the inverse-variance method for the fixed-effects model and the DerSimonian–Laird random-effects model [50]. Continuous outcomes were measured using the standardized mean difference (MD), and 95% CIs were calculated using the inverse-variance method [51]. Statistical significance was set at *p* < 0.05. Heterogeneity was assessed using the I^2^ statistic and the Cochran’s Q test [52]. An I^2^ value of >50% and a Cochran’s Q test *p*-value of <0.1 indicated significant heterogeneity. Publication bias was evaluated using funnel plots and Egger’s test [53]. Data were managed and analyzed using the “metafor” and “meta” packages of R software (R Core Team (2023) R: A Language and Environment for Statistical Computing. R Foundation for Statistical Computing, Vienna, Austria).

## 3. Results

### 3.1. Search Results

Fifty-six studies were screened initially. After removing duplicate entries, 20 records were excluded based on title or abstract screening. Further application of the inclusion and exclusion criteria resulted in the exclusion of an additional 10 studies (Figure 1). Thus, nine studies were included in the meta-analysis.

### 3.2. Characteristics of the Included Studies

Table 1 summarizes the study’s characteristics. Two trials compared IVIs of ranibizumab and SML and IVIs of ranibizumab alone; three studies compared IVIs of bevacizumab and SML and IVIs of bevacizumab alone; and four studies compared IVIs of aflibercept and SML and IVIs of aflibercept alone. These studies included 38–98 eyes and were published between 2018 and 2022. The mean age of the participants was 61.62 (range: 57.55–64.5) years, and 54.3% of the total participants were men (range: 42.5–65.8%). The study population included patients with center-involved DME, treatment-naïve centers involving DME, and refractory DME. The pre-operative BCVA, CMT, and HbA1c ratio were 0.41 (range: 0.35–0.755) logMAR, 428.92 (range: 362.6–503.69) μm, and 7.43% (range: 6.88–8.45%), respectively. Seven studies used SML with a wavelength of 577 nm, one study used 810 nm, and another study used 532 nm. Six studies mentioned complications or adverse events related to anti-VEGF therapy and SML use. No detectable scars were observed in SML. There were no major injection-related ocular or systemic adverse effects, except for some cases of subconjunctival hemorrhage or low-grade ocular inflammation. Detailed treatment protocols and complications of the included studies are presented in Table S4.

### 3.3. BCVA

The 3-month logMAR BCVA outcomes were assessed in four studies involving 259 patients. Table 2 presents the BCVA outcomes. No significant difference was observed between the IVI + SML and IVI only groups (random-effects; MD, −0.01; 95% CI: −0.12 to 0.10; *p*-value = 0.02). Similarly, no significant difference was observed between the IVI + SML and IVI only groups in terms of the 6-month logMAR BCVA, which was evaluated in four studies involving 206 patients (random-effects; MD, −0.02; 95% CI: −0.10 to 0.06; *p*-value = 0.01). However, the logMAR BCVA was significantly higher in the IVI + SML group than that in the IVI only group at the 12-month follow-up visit, as reported in the six studies involving 342 patients (random-effects: MD, −0.05; 95% CI: −0.10 to −0.01; *p*-value = 0.28). In summary, the logMAR BCVA at the 3- and 6-month follow-up visits following combination therapy with IVI + SML did not differ significantly compared with that following treatment with IVI alone. However, the logMAR BCVA at the 12-month follow-up visit was significantly better in the IVI + SML group, suggesting the potential long-term benefits of combination therapy for DME treatment.

### 3.4. CMT

Analysis of the 3-month CMT outcomes of five studies involving 313 patients revealed no significant difference between the IVI +SML and IVI-only groups (random-effects; MD, −2.66; 95% CI: −20.67 to 15.35; *p*-value = 0.02). Table 2 presents the CMT outcomes.

Similarly, no significant difference was found between the 6-month CMT outcomes of the IVI + SML and IVI only groups assessed in five studies with 260 patients (random-effects; MD, −3.69; 95% CI: −22.59 to 15.22; *p*-value = 0.04). However, the CMT was significantly lower in the IVI + SML group than that in the IVI only group in the seven studies with 396 patients that investigated the 12-month CMT outcomes (random-effects; MD, −18.27; 95% CI: −27.36 to −9.18; *p*-value = 0.20). In summary, the combination therapy of IVI + SML did not demonstrate significant differences in CMT at the 3- and 6-month follow-up visits compared with IVI-only therapy. However, CMT was significantly lower at the 12-month follow-up visit in the IVI + SML group, indicating the potential long-term benefits of combination therapy for DME treatment.

### 3.5. Total Number of IVIs

The total number of IVIs was assessed in five studies involving 302 patients (Table 2). The number of IVIs in the IVI + SML group was significantly lower than that in the IVI only group (random-effects; MD, −2.22; 95% CI: −3.13 to −1.31; *p*-value < 0.01). In summary, the combination therapy of IVI + SML was associated with a significantly lower number of IVIs than that with IVIs alone. These findings suggest that combination therapy may reduce the frequency of injections, potentially leading to a decreased risk of injection-related complications and improved patient compliance with DME treatment.

### 3.6. Subgroup Analyses, Sensitivity Analyses, and Publication Bias

A subgroup analysis was conducted to assess the impact of different SML wavelengths on the treatment outcomes, especially the 532, 577, and 810 nm wavelengths. The results revealed that patients treated with an SML wavelength of 577 nm demonstrated better outcomes with respect to the 12-month logMAR BCVA, 12-month CMT, and total number of IVIs. In contrast, no significant differences in the outcomes for logMAR BCVA, CMT, or the total number of IVIs were observed at any time point with SML wavelengths of 532 and 810 nm. In summary, the subgroup analysis indicated that the choice of SML wavelength may play a role in the effectiveness of combination therapy for DME treatment. Specifically, the 577 nm SML wavelength appeared to be associated with improvements in the 12-month logMAR BCVA, 12-month CMT, and the total number of IVIs required (Table 3). 

Another subgroup analysis was performed to evaluate different anti-VEGF agents, including ranibizumab, bevacizumab, and aflibercept. However, no significant differences were found, indicating that none of these drugs was more effective than the others (Figure S3).

No significant alterations in any of the evaluated outcomes were observed in the sensitivity analysis that excluded studies that used different treatment protocols (Figure S2). Furthermore, no publication bias was detected using funnel plots or Egger’s test.

## 4. Discussion

This meta-analysis aimed to evaluate whether combining SML therapy with anti-VEGF injections could reduce the treatment burden of frequent anti-VEGF injections and improve functional and structural outcomes in patients with DME. The present study demonstrated that SML therapy in combination with anti-VEGF injections reduces the total number of injections required, improves VA, and reduces CMT in patients with DME. These results support the assumption that SML therapy in combination with anti-VEGF injections may be beneficial in patients with DME, especially in those who cannot bear the cost and inconvenience associated with frequent injections. Although no significant differences were observed at the 3- and 6-month follow-up visits, greater effectiveness at the 12-month follow-up visit was noted with combination therapy.

SML inhibits the activity of retinal glial cells and induces downregulation of inflammatory retinal processes and a decrease in the release of VEGF to reestablish the structural and vascular integrity of the retina. SML requires a few months to demonstrate its therapeutic effect [33]; in contrast, anti-VEGF injections have a rapid effect, such as reducing vessel permeability and restoring macular integrity, that can be observed within days to weeks [28,30,31,54]. This may explain the late improvement at the 12-month follow-up visit in the combination group. In addition, most of the included trials involved patients who received three IVIs as a loading dose; therefore, the treatment effects may not have been fully expressed during the initial months. Furthermore, the relatively short follow-up duration of 6 months and varying timing of SML administration in the included studies may have influenced its effects. Nevertheless, the results of the present study may be reasonable given that SML requires several months to fully present its benefits. However, further studies must be conducted to determine whether combination therapy can maintain its efficacy for more than 12 months.

In a previous systemic review, Gawęcki [55] reported that the combination of SML with anti-VEGF injections for DME was associated with a reduction in the number of IVIs required compared with that following anti-VEGF monotherapy, while achieving similar functional and morphological outcomes. However, the number of studies was limited. In a randomized clinical trial by Furashova et al. [41], combining intravitreal ranibizumab injections with SML for DME treatment reduced the number of IVIs required. However, the trial revealed no significant variations in VA and CMT between combination therapy and anti-VEGF monotherapy at the end of the study. Incorporating SML therapy into the treatment regimen with anti-VEGF injections decreased the overall number of IVIs required during the follow-up period in the present study. Collectively, these results imply that SML combined with anti-VEGF therapy has the potential to improve functional and morphological outcomes and reduce the requirement for additional injections.

SML was first introduced by Friberg and Karatza in 1997 to treat DME using an 810 nm diode laser [56]. Different wavelength selections for retinal photocoagulation have been investigated subsequently. The use of yellow light with a 577 nm wavelength micropulse laser has gained popularity in the treatment of DME owing to its safety. Xanthophyll, primarily located in the macula, absorbs yellow light to a minimal extent, making it a suitable treatment choice [32,33,34]. Vujosevic et al. [24] compared the use of yellow 577 nm and infrared 810 nm wavelength SML in patients with mild DME (CMT < 400 μm). They reported that the 577 nm SML demonstrated slightly better results than those with the 810 nm SML, although there were no significant differences. However, some studies have suggested that the 810 nm SML has a markedly wider therapeutic range and safety margin than the 577 nm SML [57]. A review also reported that the use of SML with an 810 nm wavelength has a broad therapeutic window and well-investigated treatment parameters based on extensive clinical experience [22].

Notably, using wavelengths of <810 nm may increase the risk of retinal burns owing to the higher energy levels and varying tissue absorption characteristics [22]. Given that the yellow laser system is more readily available at present, fixed laser parameter sets should be established to prevent unintended retinal damage. A subgroup analysis of the 577 nm wavelength SML revealed a reduction in CMT, an improvement in VA, and a decrease in the total number of anti-VEGF injections required at the 12-month follow-up visit in the present study. However, most included studies in this subgroup analysis use 577 nm wavelength SML, which requires special attention in the interpretation. Despite yielding positive findings, the parameters of the SML settings used in the included studies were inconsistent. Further studies should be conducted to establish the laser parameters of the 577 nm SML and its related efficacy in combination therapy with anti-VEGF injections. Most of the included studies administered SML following three monthly anti-VEGF injections. The average CMT in these studies that reported the 3-month results was 319.80 (range: 283.37–455.24) μm [37,38,39,43,45]. This indicates that the patients could potentially be classified as having mild DME, except in the study by Akhlaghi et al. [37], which included patients with refractory DME. The CMT at 3 months was >400 μm in these patients, despite administering a loading dose of anti-VEGF injections. In a previous study by Mansouri et al. [27], SML was more effective in treating DME when the CMT was <400 μm, especially in patients with mild to moderate DME. Severe macular edema may dilute the concentration of edema-reducing cytokines induced by SML therapy and affect the distribution of laser energy throughout the retina and retinal pigment epithelium. Alternatively, anti-VEGF therapy can be initially used to reduce CMT, followed by the addition of SML to decrease the number of required injections. However, additional details regarding SML, including the wavelength, settings, timing of intervention, and treatment frequency, must be clarified in future studies.

Current anti-VEGF medications mainly include ranibizumab, bevacizumab, and aflibercept. Subgroup analysis of different anti-VEGF treatments for DME showed no statistical difference in BCVA, CMT, or the total number of required injections. Newly developed anti-VEGF medications, such as brolucizumab and faricimab, are also being used to treat DME. Future clinical trials may explore the benefits of combining anti-VEGF therapy with SML therapy, potentially leading to new findings.

The present study had some limitations. First, the small number of trials included in the present study may limit the applicability of the findings. Second, although most studies had a follow-up period of 12 months, there were differences in the follow-up duration among the included studies. Third, although all studies included patients with DME, subtle differences, such as some studies involving treatment-naïve patients, patients with refractory disease, and patients with mild DME (CMT < 350 μm), were present. Fourth, the use of anti-VEGF agents was not restricted. The included studies primarily used ranibizumab, bevacizumab, and aflibercept. Treatment efficacy may differ among these medications. Fifth, although no publication bias was detected using funnel plots and Egger’s test, the findings of this study should be interpreted with caution. The limited number of studies included in this meta-analysis could have affected the assessment of publication bias. Lastly, an important limitation of the present study was the variation in treatment protocols among the included trials, including differences in the length, settings, intervention time, and frequency of SML. However, most trials used a regimen that involved the application of SML after three monthly anti-VEGF injections. These variations may have contributed to heterogeneity in the meta-analysis and limited the reliability and validity of the conclusions of the present study.

In conclusion, the combination of SML therapy with anti-VEGF injections may reduce the total number of injections required, improve VA, and reduce CMT at the 12-month follow-up visit. This study suggests initiating anti-VEGF therapy in patients with DME with a loading dose (usually 3 injections) until macular edema is decreased, followed by the initiation of SML therapy. However, treatment protocols for combination therapy remain unclear. Further studies should be conducted to investigate the long-term efficacy and safety of combination therapies in patients with DME.

## Figures and Tables

**Table 1:** Characteristics of included studies [36,37,38,39,40,42,43,44,45].

Study	Trial Design	Population	Eyes (N)	Age (years)	Male (%)	Pre-BCVA (LogMAR)	Pre-CMT (μm)	HbA1c (%)	Anti-VEGF	SML Wavelength (nm)	Outcome	Follow-Up Duration (Months)	Intervention	Comparator
Moisseiev (2018) [36]	Cohort study	Center-involved DME	38(19/19)	65.3/63.3	63.2/68.4	0.29/0.41	316.8/408.4	NA	R	577	BCVA, CMT, IVI total number	19.1/23.2	IVI-R+SML	IVI-R
Akhlaghi (2019) [37]	RCT	Refractory DME	42(21/21)	60.86 *	47.6 *	0.81/0.70	513/494.38	NA	B	810	BCVA, CMT	NA	IVI-B+SML	IVI-B
Khattab (2019) [38]	RCT	Center-involved DME	54(27/27)	59.4/55.7	40.7/59.3	NA ***	457.1/462	NA	A	577	BCVA, CMT, CS	18	IVI-A+SML	IVI-A
Abouhussein (2020) [39]	RCT	Treatment-naïve center involved DME	40(20/20)	60.4/59.5	45/40	0.76/0.70	469.6/457.9	8.7/8.2	A	577	BCVA, CMT, IVI total number	12	IVI-A+SML	IVI-A
Kanar (2020) [40]	RCT	Treatment-naïve center involved DME	56(28/28)	63.42/62.64	54/57	0.40/0.38	466.07/451.28	7.97/8.02	A	577	BCVA, CMT, IVI total number, SFCT	12	IVI-A+SML	IVI-A
Matri (2021) [42]	Cohort study	Treatment-naïve center involved DME	98(49/49)	67.7/61.3	59.38/64.52 **	0.692/0.598	479.1/359.9	7.70/7.60	B	577	BCVA, CMT, IVI total number	12	IVI-B+SML	IVI-B
Altınel (2021) [43]	Cohort study	Center-involved DME	80(40/40)	60.55/59.83	57.5/55	0.38/0.39	379.2/384.68	6.94/6.89	B	577	BCVA, CMT, IVI total number	11.48/11.1	IVI-B+SML	IVI-B
Koushan (2022) [44]	RCT	Center-involved DME	30(15/15)	59.8/58.8	66.7/46.7	0.36/0.38	457.8/433.4	NA	A	532	BCVA, CMT, IVI total number	12	IVI-A+SML	IVI-A
Bıçak (2022) [45]	Cohort study	Center-involved DME (CMT≤350 μm)	97(52/45)	62.4/61.6	53.8/44.4	0.43/0.41	426.6/406.0	6.91/6.85	R	577	BCVA, CMT, IVI total number, MV	9.25/9.29	IVI-R+SML	IVI-R

Legend: DME, diabetic macular edema; N, number (intervention/comparator); BCVA, best corrected visual acuity; CMT, central macular thickness; R, ranibizumab; B, bevacizumab; A, aflibercept; IVI, Intravitreal injection; CS, contrast sensitivity; SCFT, subfoveal choroidal thickness; MV, macular volumes; NA, not applicable. * The study included patients with DME in both eyes and they were randomly divided into two groups: one that received the intervention and another that served as the comparator group. ** The study did not provide specific eye counts; instead, we calculated the percentage based on the number of individuals. *** The study used as the parameter for measuring visual acuity, not logMAR.

**Table 2:** Summary table of meta-analysis results for each outcome.

Outcome	Follow-up period	Studies Number (N)	Patients Number (N)	Measurement (95% CIs)	Cochran Q *p*-Value	I^2^ (%)
logMAR BCVA	3 months	4	259	random−effects; MD, −0.01 (−0.12 to 0.10)	0.02	69%
	6 months	4	206	random−effects; MD, −0.02 (−0.10 to 0.06)	0.01	72%
	12 months	6	342	random−effects; MD, −0.05 (−0.10 to −0.01)	0.28	20%
Central Macular Thickness (CMT)	3 months	5	313	random−effects; MD, −2.66 (−20.67 to 15.35)	0.02	65%
	6 months	5	260	random−effects; MD, −3.69 (−22.59 to 15.22)	0.04	60%
	12 months	7	396	random−effects; MD, −18.27 (−27.36 to −9.18)	0.20	29%
Total Number of IVI	Overall	5	302	random−effects; MD, −2.22 (−3.13 to −1.31)	<0.01	84%

Legend: BCVA, best corrected visual acuity; OR, odds ratio; MD, mean difference; CIs, confident intervals; N, number; IVI, intravitreal Injections.

**Table 3:** Subgroup analysis of outcomes for different wavelengths of subthreshold micropulse laser.

Outcome	Follow-Up Period/Subgroup	Studies Number (N)	Patients Number (N)	Measurement (95% CIs)	Cochran Q *p*-Value	I^2^ (%)
logMAR BCVA	3 months–Overall	4	259	random−effects; MD, −0.01 (−0.12 to 0.10)	0.02	69%
	3 months–810 nm	1	42	MD, −0.17 (−0.35 to 0.01)	-	-
	3 months–577 nm	3	217	random−effects; MD, 0.03 (−0.07 to 0.13)	0.06	63%
	6 months–Overall	4	206	random−effects; MD, −0.02 (−0.10 to 0.06)	0.01	72%
	6 months–577 nm	3	176	random−effects; MD, 0.00 (−0.09 to 0.11)	0.02	75%
	6 months–532 nm	1	30	MD, −0.11 (−0.23 to 0.01)	-	-
	12 months–Overall	6	342	random−effects; MD, −0.05 (−0.10 to −0.01)	0.28	20%
	12 months–577 nm	5	312	random−effects; MD, −0.05 (−0.10 to −0.01)	0.28	20%
	12 months–532 nm	1	15	MD, −0.10 (−0.23 to 0.03)	0.93	0%
Central Macular Thickness (CMT)	3 months–Overall	5	313	random−effects; MD, −2.66 (−20.67 to 15.35)	0.02	65%
	3 months–810 nm	1	42	MD, −94.28 (−168.80 to −19.76)	-	-
	3 months–577 nm	4	271	random−effects; MD, 2.56 (−10.35 to 15.48)	0.17	40%
	6 months–Overall	5	260	random−effects; MD, −3.69 (−22.59 to 15.22)	0.04	60%
	6 months–577 nm	4	230	random−effects; MD, −3.60 (−25.76 to 18.56)	0.02	69%
	6 months–532 nm	1	30	MD, −7.30 (−48.06 to 33.46)	-	-
	12 months–Overall	7	396	random−effects; MD, −18.27 (−27.36 to −9.18)	0.20	29%
	12 months–577 nm	6	366	random−effects; MD, −19.90 (−29.30 to −10.60)	0.22	29%
	12 months–532 nm	1	30	MD, 1.20 (−27.79 to 30.19)	-	-
Total Number of IVI	Overall	5	302	random−effects; MD, −2.22 (−3.13 to −1.31)	<0.01	84%
	577 nm	4	272	random−effects; MD, −2.38 (−3.33 to −1.42)	<0.01	88%
	532 nm	1	30	MD, −0.60 (−3.07 to 1.87)	-	-

Legend: BCVA, best corrected visual acuity; OR, odds ratio; MD, mean difference; CIs, confident intervals; N, number; IVI, intravitreal injections.

## Data Availability

Data are contained within the article or Supplementary Materials.

## Supplementary Materials

The following supporting information can be downloaded at: https://www.mdpi.com/article/10.3390/jcm13164782/s1, Table S1. PRISMA Checklist. Table S2. MOOSE Checklist. Table S3. Search strategy. Table S4. Detailed treatment protocols and complications of included studies. Figure S1. Assessment of risk of bias. Figure S2. Sensitivity analysis of outcomes. Figure S3. Subgroup Analysis of Outcomes for Different Anti-VEGF medications.

## References

[1] Lee R., Wong T.Y., Sabanayagam C. (2015). Epidemiology of diabetic retinopathy, diabetic macular edema and related vision loss. Eye Vis..

[2] Bahrami B., Zhu M., Hong T., Chang A. (2016). Diabetic macular oedema: Pathophysiology, management challenges and treatment resistance. Diabetology.

[3] Das A., McGuire P.G., Rangasamy S. (2015). Diabetic macular edema: Pathophysiology and novel therapeutic targets. Ophthalmology.

[4] Al Shamsi H., Ghazi N.G. (2012). Diabetic macular edema: New trends in management. Expert Rev. Clin. Pharmacol..

[5] Early Treatment Diabetic Retinopathy Study Research Group (1985). Photocoagulation for diabetic macular edema. Early Treatment Diabetic Retinopathy Study report number 1. Arch. Ophthalmol..

[6] Hudson C., Flanagan J.G., Turner G.S., Chen H.C., Young L.B., McLeod D. (1998). Influence of laser photocoagulation for clinically significant diabetic macular oedema (DMO) on short-wavelength and conventional automated perimetry. Diabetologia.

[7] Schatz H., Madeira D., McDonald H.R., Johnson R.N. (1991). Progressive enlargement of laser scars following grid laser photocoagulation for diffuse diabetic macular edema. Arch. Ophthalmol..

[8] Lewis H., Schachat A.P., Haimann M.H., Haller J.A., Quinlan P., von Fricken M.A., Fine S.L., Murphy R.P. (1990). Choroidal neovascularization after laser photocoagulation for diabetic macular edema. Ophthalmology.

[9] Guyer D.R., D’Amico D.J., Smith C.W. (1992). Subretinal fibrosis after laser photocoagulation for diabetic macular edema. Am. J. Ophthalmol..

[10] Elman M.J., Aiello L.P., Beck R.W., Bressler N.M., Bressler S.B., Edwards A.R., Ferris F.L., Friedman S.M., Glassman A.R., Diabetic Retinopathy Clinical Research Network (2010). Randomized trial evaluating ranibizumab plus prompt or deferred laser or triamcinolone plus prompt laser for diabetic macular edema. Ophthalmology.

[11] Mitchell P., Bandello F., Schmidt-Erfurth U., Lang G.E., Massin P., Schlingemann R.O., Sutter F., Simader C., Burian G., Gerstner O. (2011). The RESTORE study: Ranibizumab monotherapy or combined with laser versus laser monotherapy for diabetic macular edema. Ophthalmology.

[12] Nguyen Q.D., Brown D.M., Marcus D.M., Boyer D.S., Patel S., Feiner L., Gibson A., Sy J., Rundle A.C., Hopkins J.J. (2012). Ranibizumab for diabetic macular edema: Results from 2 phase III randomized trials: RISE and RIDE. Ophthalmology.

[13] Wells J.A., Glassman A.R., Ayala A.R., Jampol L.M., Aiello L.P., Antoszyk A.N., Arnold-Bush B., Baker C.W., Bressler N.M., Diabetic Retinopathy Clinical Research Network (2015). Aflibercept, bevacizumab, or ranibizumab for diabetic macular edema. N. Engl. J. Med..

[14] Elman M.J., Ayala A., Bressler N.M., Browning D., Flaxel C.J., Glassman A.R., Jampol L.M., Stone T.W., Diabetic Retinopathy Clinical Research Network (2015). Intravitreal ranibizumab for diabetic macular edema with prompt versus deferred laser treatment: 5-year randomized trial results. Ophthalmology.

[15] Flaxel C.J., Adelman R.A., Bailey S.T., Fawzi A., Lim J.I., Vemulakonda G.A., Ying G.S. (2020). Diabetic Retinopathy Preferred Practice Pattern^®^. Ophthalmology.

[16] Schmidt-Erfurth U., Garcia-Arumi J., Bandello F., Berg K., Chakravarthy U., Gerendas B.S., Jonas J., Larsen M., Tadayoni R., Loewenstein A. (2017). Guidelines for the Management of Diabetic Macular Edema by the European Society of Retina Specialists (EURETINA). Ophthalmologica.

[17] Bakri S.J., Wolfe J.D., Regillo C.D., Flynn H.W., Wykoff C.C. (2019). Evidence-Based Guidelines for Management of Diabetic Macular Edema. J. Vitr. Dis..

[18] Chen J.T., Chen L.J., Chen S.N., Chen W.L., Cheng C.K., Hsu S.M., Sheu S.J., Wu W.C., Yang C.H., Yang C.M. (2020). Management of diabetic macular edema: Experts’ consensus in Taiwan. Jpn. J. Ophthalmol..

[19] Virgili G., Parravano M., Evans J.R., Gordon I., Lucenteforte E. (2017). Anti-vascular endothelial growth factor for diabetic macular oedema: A network meta-analysis. Cochrane Database Syst. Rev..

[20] Sampat K.M., Garg S.J. (2010). Complications of intravitreal injections. Curr. Opin. Ophthalmol..

[21] Falavarjani K.G., Nguyen Q.D. (2013). Adverse events and complications associated with intravitreal injection of anti-VEGF agents: A review of literature. Eye.

[22] Luttrull J.K., Dorin G. (2012). Subthreshold diode micropulse laser photocoagulation (SDM) as invisible retinal phototherapy for diabetic macular edema: A review. Curr. Diabetes Rev..

[23] Vujosevic S., Bottega E., Casciano M., Pilotto E., Convento E., Midena E. (2010). Microperimetry and fundus autofluorescence in diabetic macular edema: Subthreshold micropulse diode laser versus modified early treatment diabetic retinopathy study laser photocoagulation. Retina.

[24] Vujosevic S., Martini F., Longhin E., Convento E., Cavarzeran F., Midena E. (2015). Subthreshold micropulse yellow laser versus subthreshold micropulse infrared laser in center-involving diabetic macular edema: Morphologic and functional safety. Retina.

[25] Luttrull J.K., Sramek C., Palanker D., Spink C.J., Musch D.C. (2012). Long-term safety, high-resolution imaging, and tissue temperature modeling of subvisible diode micropulse photocoagulation for retinovascular macular edema. Retina.

[26] Luttrull J.K., Sinclair S.H. (2014). Safety of transfoveal subthreshold diode micropulse laser for fovea-involving diabetic macular edema in eyes with good visual acuity. Retina.

[27] Mansouri A., Sampat K.M., Malik K.J., Steiner J.N., Glaser B.M. (2014). Efficacy of subthreshold micropulse laser in the treatment of diabetic macular edema is influenced by pre-treatment central foveal thickness. Eye.

[28] Lavinsky D., Cardillo J.A., Melo L.A.S., Dare A., Farah M.E., Belfort R. (2011). Randomized clinical trial evaluating mETDRS versus normal or high-density micropulse photocoagulation for diabetic macular edema. Investig. Ophthalmol. Vis. Sci..

[29] Nakamura Y., Mitamura Y., Ogata K., Arai M., Takatsuna Y., Yamamoto S. (2010). Functional and morphological changes of macula after subthreshold micropulse diode laser photocoagulation for diabetic macular oedema. Eye.

[30] Sivaprasad S., Sandhu R., Tandon A., Sayed-Ahmed K., McHugh D.A. (2007). Subthreshold micropulse diode laser photocoagulation for clinically significant diabetic macular oedema: A three-year follow up. Clin. Exp. Ophthalmol..

[31] Chen G., Tzekov R., Li W., Jiang F., Mao S., Tong Y. (2016). Subthreshold micropulse diode laser versus conventional laser photocoagulation for diabetic macular edema: A meta-analysis of randomized controlled trials. Retina.

[32] Gawęcki M. (2019). Micropulse Laser Treatment of Retinal Diseases. J. Clin. Med..

[33] Mainster M.A. (1986). Wavelength selection in macular photocoagulation. Tissue optics, thermal effects, and laser systems. Ophthalmology.

[34] Mainster M.A. (1999). Decreasing retinal photocoagulation damage: Principles and techniques. Semin. Ophthalmol..

[35] Sabal B., Teper S., Wylęgała E. (2022). Subthreshold micropulse laser for diabetic macular edema: A review. J. Clin. Med..

[36] Moisseiev E., Abbassi S., Thinda S., Yoon J., Yiu G., Morse L.S. (2018). Subthreshold micropulse laser reduces anti-VEGF injection burden in patients with diabetic macular edema. Eur. J. Ophthalmol..

[37] Akhlaghi M., Dehghani A., Pourmohammadi R., Asadpour L., Pourazizi M. (2019). Effects of subthreshold diode micropulse laser photocoagulation on treating patients with refractory diabetic macular edema. J. Curr. Ophthalmol..

[38] Khattab A.M., Hagras S.M., AbdElhamid A., Torky M.A., Awad E.A., Abdelhameed A.G. (2019). Aflibercept with adjuvant micropulsed yellow laser versus aflibercept monotherapy in diabetic macular edema. Graefe’s Arch. Clin. Exp. Ophthalmol..

[39] Abouhussein M.A., Gomaa A.R. (2020). Aflibercept plus micropulse laser versus aflibercept monotherapy for diabetic macular edema: 1-year results of a randomized clinical trial. Int. Ophthalmol..

[40] Kanar H.S., Arsan A., Altun A., Akı S.F., Hacısalihoglu A. (2020). Can subthreshold micropulse yellow laser treatment change the anti-vascular endothelial growth factor algorithm in diabetic macular edema? A randomized clinical trial. Indian J. Ophthalmol..

[41] Furashova O., Strassburger P., Becker K.A., Engelmann K. (2020). Efficacy of combining intravitreal injections of ranibizumab with micropulse diode laser versus intravitreal injections of ranibizumab alone in diabetic macular edema (ReCaLL): A single center, randomised, controlled, non-inferiority clinical trial. BMC Ophthalmol..

[42] El Matri L., Chebil A., El Matri K., Falfoul Y., Chebbi Z. (2021). Subthreshold micropulse laser adjuvant to bevacizumab versus bevacizumab monotherapy in treating diabetic macular edema: One- year- follow-up. Ther. Adv. Ophthalmol.

[43] Altınel M.G., Acikalin B., Alis M.G., Demir G., Mutibayraktaroglu K.M., Totuk O.M.G., Ardagil A. (2021). Comparison of the efficacy and safety of anti-VEGF monotherapy versus anti-VEGF therapy combined with subthreshold micropulse laser therapy for diabetic macular edema. Lasers Med. Sci..

[44] Koushan K., Eshtiaghi A., Fung P., Berger A.R., Chow D.R. (2022). Treatment of diabetic macular edema with aflibercept and micropulse laser (DAM study). Clin. Ophthalmol..

[45] Bıçak F., Kayıkçıoğlu Ö.R., Altınışık M., Doğruya S., Kurt E. (2022). Efficacy of subthreshold micropulse laser combined with ranibizumab in the treatment of diabetic macular edema. Int. Ophthalmol..

[46] Liberati A., Altman D.G., Tetzlaff J., Mulrow C., Gøtzsche P.C., Ioannidis J.P.A., Clarke M., Devereaux P.J., Kleijnen J., Moher D. (2009). The PRISMA statement for reporting systematic reviews and meta-analyses of studies that evaluate health care interventions: Explanation and elaboration. J. Clin. Epidemiol..

[47] Stroup D.F., Berlin J.A., Morton S.C., Olkin I., Williamson G.D., Rennie D., Moher D., Becker B.J., Sipe T.A., Thacker S.B. (2000). Meta-analysis of observational studies in epidemiology: A proposal for reporting. Meta-analysis Of Observational Studies in Epidemiology (MOOSE) group. JAMA.

[48] Stang A. (2010). Critical evaluation of the Newcastle-Ottawa scale for the assessment of the quality of nonrandomized studies in meta-analyses. Eur. J. Epidemiol..

[49] Cumpston M., Li T., Page M.J., Chandler J., Welch V.A., Higgins J.P., Thomas J. (2019). Updated guidance for trusted systematic reviews: A new edition of the Cochrane Handbook for Systematic Reviews of Interventions. Cochrane Database Syst. Rev..

[50] Hartung J., Knapp G. (2001). A refined method for the meta-analysis of controlled clinical trials with binary outcome. Stat. Med..

[51] Higgins J.P., Whitehead A., Turner R.M., Omar R.Z., Thompson S.G. (2001). Meta-analysis of continuous outcome data from individual patients. Stat. Med..

[52] Higgins J.P.T., Thompson S.G., Deeks J.J., Altman D.G. (2003). Measuring inconsistency in meta-analyses. BMJ.

[53] van Aert R.C.M., Wicherts J.M., van Assen M.A.L.M. (2019). Publication bias examined in meta-analyses from psychology and medicine: A meta-meta-analysis. PLoS ONE.

[54] Frizziero L., Calciati A., Midena G., Torresin T., Parrozzani R., Pilotto E., Midena E. (2021). Subthreshold micropulse laser modulates retinal neuroinflammatory biomarkers in diabetic macular edema. J. Clin. Med..

[55] Gawęcki M. (2021). Subthreshold diode micropulse laser combined with intravitreal therapy for macular edema-a systematized review and critical approach. J. Clin. Med..

[56] Friberg T.R., Karatza E.C. (1997). The treatment of macular disease using a micropulsed and continuous wave 810-nm diode laser. Ophthalmology.

[57] Chang D.B., Luttrull J.K. (2020). Comparison of subthreshold 577 and 810 nm micropulse laser effects on heat-shock protein activation kinetics: Implications for treatment efficacy and safety. Transl. Vis. Sci. Technol..

