# Supplementary material for: Optimizing Diabetic Macular Edema Treatment: A Meta-Analysis of Subthreshold Micropulse Laser and Anti-Vascular Endothelial Growth Factor Combination Therapy
Source: J Clin Med. 2024 Aug 14;13(16):4782. doi: 10.3390/jcm13164782 (PMC11355810; doi:10.3390/jcm13164782)

Optimizing Diabetic Macular Edema Treatment: A Meta-Analysis of Subthreshold Micropulse Laser and Anti-Vascular Endothelial Growth Factor Combination Therapy

Table S1. PRISMA Checklist

Table S2. MOOSE Checklist

Table S3. Search strategy

Table S4. Detailed treatment protocols and complications of included studies

Figure S1. Assessment of risk of bias

Figure S2. Sensitivity analysis of outcomes

Figure S3. Subgroup Analysis of Outcomes for Different Anti-VEGF medications

Table S1. PRISMA Checklist

Section/topic	Item No	Checklist item	Reported on page No
**Title**			
Title	1	Identify the report as a systematic review, meta-analysis, or both	3
**Abstract**			
Structured summary	2	Provide a structured summary including, as applicable, background, objectives, data sources, study eligibility criteria, participants, interventions, study appraisal and synthesis methods, results, limitations, conclusions and implications of key findings, systematic review registration number	23
**Introduction**			
Rationale	3	Describe the rationale for the review in the context of what is already known	4
Objectives	4	Provide an explicit statement of questions being addressed with reference to participants, interventions, comparisons, outcomes, and study design (PICOS)	4
**Methods**			
Protocol and registration	5	Indicate if a review protocol exists, if and where it can be accessed (such as web address), and, if available, provide registration information including registration number	5
Eligibility criteria	6	Specify study characteristics (such as PICOS, length of follow-up) and report characteristics (such as years considered, language, publication status) used as criteria for eligibility, giving rationale	5
Information sources	7	Describe all information sources (such as databases with dates of coverage, contact with study authors to identify additional studies) in the search and date last searched	6
Search	8	Present full electronic search strategy for at least one database, including any limits used, such that it could be repeated	6
Study selection	9	State the process for selecting studies (that is, screening, eligibility, included in systematic review, and, if applicable, included in the meta-analysis)	6
Data collection process	10	Describe method of data extraction from reports (such as piloted forms, independently, in duplicate) and any processes for obtaining and confirming data from investigators	6

Data items	11	List and define all variables for which data were sought (such as PICOS, funding sources) and any assumptions and simplifications made	6
Risk of bias in individual studies	12	Describe methods used for assessing risk of bias of individual studies (including specification of whether this was done at the study or outcome level), and how this information is to be used in any data synthesis	Supplementary
Summary measures	13	State the principal summary measures (such as risk ratio, difference in means).	8
Synthesis of results	14	Describe the methods of handling data and combining results of studies, if done, including measures of consistency (such as I^2 statistic) for each meta-analysis	68
Risk of bias across studies	15	Specify any assessment of risk of bias that may affect the cumulative evidence (such as publication bias, selective reporting within studies)	8
Additional analyses	16	Describe methods of additional analyses (such as sensitivity or subgroup analyses, meta-regression), if done, indicating which were pre-specified	8
**Results**			
Study selection	17	Give numbers of studies screened, assessed for eligibility, and included in the review, with reasons for exclusions at each stage, ideally with a flow diagram	9
Study characteristics	18	For each study, present characteristics for which data were extracted (such as study size, PICOS, follow-up period) and provide the citations	9
Risk of bias within studies	19	Present data on risk of bias of each study and, if available, any outcome-level assessment (see item 12).	Supplementary
Results of individual studies	20	For all outcomes considered (benefits or harms), present for each study (a) simple summary data for each intervention group and (b) effect estimates and confidence intervals, ideally with a forest plot	9
Synthesis of results	21	Present results of each meta-analysis done, including confidence intervals and measures of consistency	10
Risk of bias across studies	22	Present results of any assessment of risk of bias across studies (see item 15)	Supplementary
Additional analysis	23	Give results of additional analyses, if done (such as sensitivity or subgroup analyses, meta-regression) (see item 16)	13

**Discussion**			
Summary of evidence	24	Summarise the main findings including the strength of evidence for each main outcome; consider their relevance to key groups (such as health care providers, users, and policy makers)	13
Limitations	25	Discuss limitations at study and outcome level (such as risk of bias), and at review level (such as incomplete retrieval of identified research, reporting bias)	16
Conclusions	26	Provide a general interpretation of the results in the context of other evidence, and implications for future research	16
**Funding**			
Funding	27	Describe sources of funding for the systematic review and other support (such as supply of data) and role of funders for the systematic review	Funding part

Table S2. MOOSE Checklist

Item No	Recommendation	Reported on Page No
Reporting of background should include		
1	Problem definition	5
2	Hypothesis statement	5
3	Description of study outcome(s)	7
4	Type of exposure or intervention used	7
5	Type of study designs used	7
6	Study population	7
Reporting of search strategy should include		
7	Qualifications of searchers (eg, librarians and investigators)	6
8	Search strategy, including time period included in the synthesis and keywords	6
9	Effort to include all available studies, including contact with authors	6
10	Databases and registries searched	6
11	Search software used, name and version, including special features used (eg, explosion)	6
12	Use of hand searching (eg, reference lists of obtained articles)	6
13	List of citations located and those excluded, including justification	6
14	Method of addressing articles published in languages other than English	6
15	Method of handling abstracts and unpublished studies	6
16	Description of any contact with authors	6
Reporting of methods should include		
17	Description of relevance or appropriateness of studies assembled for assessing the hypothesis to be tested	8
18	Rationale for the selection and coding of data (eg, sound clinical principles or convenience)	Supplementary
19	Documentation of how data were classified and coded (eg, multiple raters, blinding and interrater reliability)	Supplementary
20	Assessment of confounding (eg, comparability of cases and controls in studies where appropriate)	Supplementary
21	Assessment of study quality, including blinding of quality assessors, stratification or regression on possible predictors of study results	Supplementary
22	Assessment of heterogeneity	8
23	Description of statistical methods (eg, complete description of fixed or random effects models, justification of whether the chosen models account for predictors of study results, dose-response models, or cumulative meta-analysis) in sufficient detail to be replicated	8
24	Provision of appropriate tables and graphics	8

Item No	Recommendation	Reported on Page No
Reporting of results should include		
25	Graphic summarizing individual study estimates and overall estimate	9
26	Table giving descriptive information for each study included	9
27	Results of sensitivity testing (eg, subgroup analysis)	13
28	Indication of statistical uncertainty of findings	13
Reporting of discussion should include		
29	Quantitative assessment of bias (eg, publication bias)	13
30	Justification for exclusion (eg, exclusion of non-English language citations)	Supplementary
31	Assessment of quality of included studies	Supplementary
Reporting of conclusions should include		
32	Consideration of alternative explanations for observed results	16
33	Generalization of the conclusions (ie, appropriate for the data presented and within the domain of the literature review)	16
34	Guidelines for future research	16
35	Disclosure of funding source	17

From: Stroup DF, Berlin JA, Morton SC, et al, for the Meta-analysis Of Observational Studies in Epidemiology (MOOSE) Group. Meta-analysis of Observational Studies in Epidemiology. A Proposal for Reporting. *JAMA*. 2000;283(15):2008-2012. doi: 10.1001/jama.283.15.2008.

Table S3. Search strategy

We used the search strategy outlined below to search PubMed, Embase, and Cochrane Library databases for studies related to diabetic macular edema, laser treatment, intravitreal injections, and anti-VEGF therapy:

PubMed:
(("Diabetic Macular Edema"[Mesh] OR "diabetic macular edema" OR "DME") AND ("Lasers, Solid-State"[Mesh] OR "laser treatment" OR "laser photocoagulation") AND ("Injections, Intraocular"[Mesh] OR "intravitreal injections" OR "intravitreal") AND ("Vascular Endothelial Growth Factor A"[Mesh] OR "anti-VEGF therapy" OR "bevacizumab" OR "ranibizumab" OR "aflibercept" OR "ziv-aflibercept" OR "conbercept" OR "brolucizumab"))

Embase:
('diabetic macular edema' OR DME OR 'macular edema' OR 'edema, macular') AND ('laser therapy' OR 'laser treatment' OR 'photocoagulation, laser') AND ('intravitreal injection' OR 'injection, intravitreal' OR 'intravitreal implant') AND ('vascular endothelial growth factor inhibitor' OR 'VEGF inhibitor' OR 'bevacizumab' OR 'ranibizumab' OR 'aflibercept' OR 'ziv-aflibercept' OR 'conbercept' OR 'brolucizumab')

Cochrane Library:
(("diabetic macular edema" OR DME) AND ("laser therapy" OR "laser treatment" OR "photocoagulation, laser") AND ("intravitreal injection" OR "injection, intravitreal" OR "intravitreal implant") AND ("vascular endothelial growth factor inhibitor" OR "VEGF inhibitor" OR "bevacizumab" OR "ranibizumab" OR "aflibercept" OR "ziv-aflibercept" OR "conbercept" OR "brolucizumab")).

Table S4. Detailed treatment protocols and complications of included studies

Study	Number of previous anti-VEGF injections before study		IVI protocol	SML protocol	SML sessions details	Complication or adverse effects	
	IVI+SML	IVI				IVI+SML	IVI
Moisseiev (2018) [36]	All included patients had no more than 3 prior IVI and the last injection more than 2 months prior to SML.	0*	Not mentioned	Not mentioned	Micropulse yellow laser (400 mW power, 200 µm spot size, 200 ms duration with 5% duty cycle). Between 150 and 250 spots were used to cover the macula, in a high-density fashion with no overlap. The first spot was placed away from the fovea to make sure no visible burn was achieved.	No	No
Akhlaghi (2019) [37]	The study included patients with DME in both eyes resistant to treatment with intravitreal bevacizumab, but it didn't provide exact number of previous injections.		3 monthly IVI, but the subsequent protocol is not mentioned	Not mentioned	Subthreshold diode micropulse laser photocoagulation (810 nm, 200 nm spot size, 5% duty cycle, and with power four times more than at least visible effect).	Not mentioned	Not mentioned
Khattab (2019) [38]	Not mentioned		3 monthly IVI, followed by monthly PRN injections (if CMT exceeds 250µm)	The first SML therapy was performed within one week after the 3rd injection. If the CMT was above 250µm, additional SML was administered, provided the last MPL session was conducted at least two months prior.	Micropulse yellow laser (577 nm, 400 mW power, 200 µm spot size, and 200 ms duration with 5% duty cycle). Laser was applied on clinically visible thickened macula with different number of confluent non-spacing shots in 7 × 7 grids.	None of the patients developed any major ocular or systemic adverse effects. Only low grade ocular inflammation was noted in two (13.3%) eyes in both groups, which resolved within one week with topical steroidal drops.	
Abouhussein (2020) [39]	The study included patients with treatment-naïve central DME.		3 monthly IVI, followed by monthly PRN injections	1 month after the 3rd injection and only once time.	Micropulse yellow laser (577 nm, 400 mW power, 200 µm spot size, and 200 ms	No detectable scars were observed in SML.	

				duration with 5% duty cycle). Confluent applications with no spacing were administered using 5 9 5 grid pattern over the entire edematous area including the fovea.	There were no injection-related complications except for mild subconjunctival hemorrhage at the site of injection.	
Kanar (2020) [40]	The study included patients with treatment-naïve central DME.	3 monthly IVI, followed by monthly PRN injections	1 month after the 3rd injection if CMT had decreased to 450µm or below; one or more IAI and SML applied 4 weeks after if CMT did not reach less than 450 µm. Patients who have increase of at least 20% CMT values and/or 1 line decreased at BCVA after the SML treatment, the laser procedure was repeated 2 months after any SML session.	Micropulse yellow laser (577nm, 160 µm spot size, 20 ms duration with a 5% duty cycle outside the vascular arcade, with the power titrated from 50 mW upward until a burn became slightly visible). The SMYL was then performed contiguously on the macular area using the same spot size by reducing the laser to half the power of the test burn.	Not mentioned	Not mentioned
Matri (2021) [42]	The study included patients with treatment-naive central DME.	3 monthly IVI, followed by monthly PRN injection	1 session on 1 week after the last IVI. If a patient is a poor responder** at the 16th week, MPL is performed at the 25th week; if a poor responder at the 32nd week, MPL is performed at the 41st week.	Micropulse yellow laser (577 nm, 400 mW power, 200 µm spot size, and 200 ms duration with 5% duty cycle). The number of spots was variable and MPL was applied with no spacing application of spots using a 2 x 2 or 4 x 4 treatment grid to cover the entire edematous area based on OCT.	No patient complained about scotoma. There were no laser scars on fundus photography, fundus autofluorescence nor SD-OCT.	The only ocular complication observed was subconjunctival hemorrhage in the injection site, in 12 eyes (12.2%).
Altınel (2021) [43]	The study included patients with no history of any kind of intravitreal anti-	3 monthly IVI, followed by monthly PRN injections	1 month after the loading phase of IVI if CMT had decreased to <400 µm; one or	Micropulse yellow laser (577 nm, 160 µm spot size, and 200 ms duration with 5%	No	Not mentioned

	VEGF or steroid therapy within the previous 6 months.		more IVBs were applied monthly until the CMT had decreased to <400 µm	duty cycle). The power titration was performed in the monospot micropulse mode and was started at 700 mW. The power was then increased enough to create a faint white spot in a peripheral retinal area outside the vascular arcade. At the macular zone, half of the energy level in the micropulse mode was applied with confluent laser spots.		
Koushan (2022) [44]	The study excluded patients with history of DME treatment in the preceding 4 months.	1 IVI, followed by monthly PRN injections	1 session on the same day of the first injection. The time between successive SML treatments was set at a minimum of 3 months.	Micropulse laser (532 nm, 200 µm spot size, and 20 ms duration with 10% duty cycle). Micropulsed laser will be used to create light (barely visible) test burns outside the macular area (starting at 100 mw with upward titration). The laser will then be decreased to 90% of that of the test burns. The final laser spots should not be visible. 3 x 3 pattern mode (confluent spots) over the entire macular area including the foveal center. Non-pattern repeat mode with overlapping burns is also permitted.	No	No
Bıçak (2022) [45]	The study included patients who had CMT ≤ 350 µm after three monthly	Monthly PRN injections	1 month after the loading dose and only once time	Micropulse yellow laser (577 nm, 165 µm spot size, and 200 ms duration with 5%	Not mentioned	Not mentioned

	doses of IVI for center-involving DME.			duty cycle). The power titration was performed in the monospot micropulse mode and was started at 600 mW, increased up until creating a visible white spot. The titration spot was performed in a retinal area two disc diameters from the posterior pole. Then the power was reduced to half at the macular zone using the micropulse mode. SML was applied at central 1000 micron of the macula using grid pattern. If necessary, additional focal laser was applied using confluent laser spots without space to the areas which increased retinal thickness in SD-OCT imaging outside the central 1000 micron of the macula. No laser was applied to the central fovea.		

Legend: VEGF, vascular endothelial growth factor; IVI, Intravitreal injection; SML, subthreshold micropulse laser; DME, diabetic macular edema

* In Moisseiev 2018, the first injection in the IVI group was defined as the initial injection, meaning there could be no history of prior injections in those eyes.

** Poor responder: decrease of BCVA by one line or more ($\geqslant$ 5 letters).

Figure S1. Assessment of risk of bias

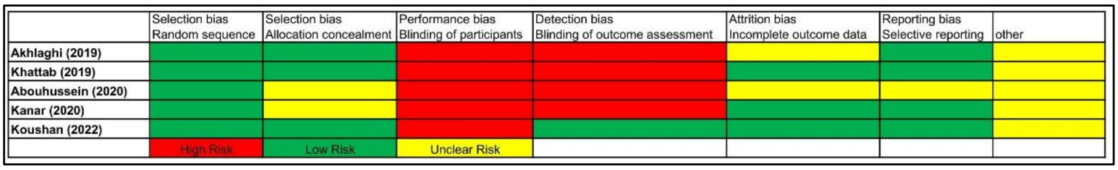

Newcastle-Ottawa Scale quality assessment scale for cohort studies

First author, year	Representativeness of the exposed cohort	Selection of the nonexposed cohort	Ascertainment of exposure	Demonstration that outcome of interest was not present at start of study	Comparability of cohorts on the basis of the design or analysis	Assessment of outcome	Was follow-up long enough for outcomes to occur	Adequacy of follow up of cohorts
Moisseiev (2018)	✸		✸		✸	✸		
Matri (2021)	✸		✸	✸	✸	✸		
Altınel (2021)	✸	✸	✸	✸	✸	✸		
Bıçak (2022)	✸	✸	✸	✸		✸		✸

Figure S2. Sensitivity analysis of outcomes

CMT_3-month_exclude different protocol

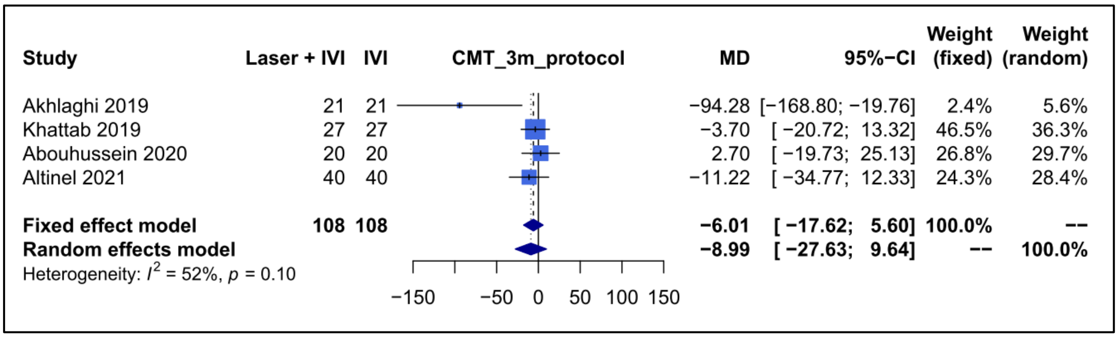

CMT_6-month_exclude different protocol

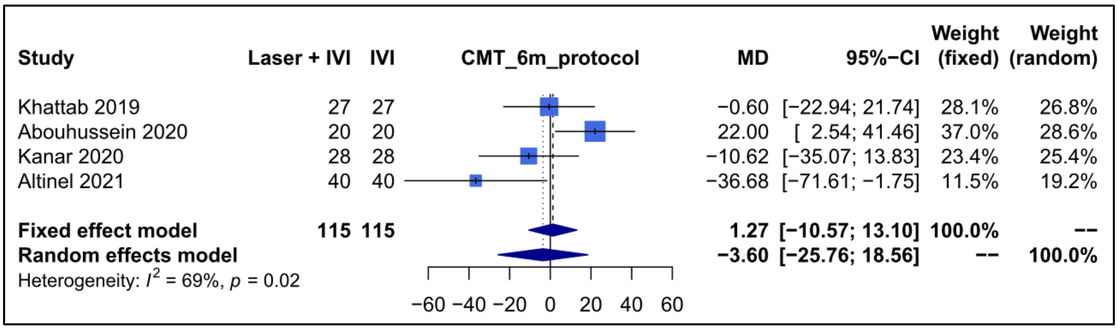

CMT_12-month_exclude different protocol

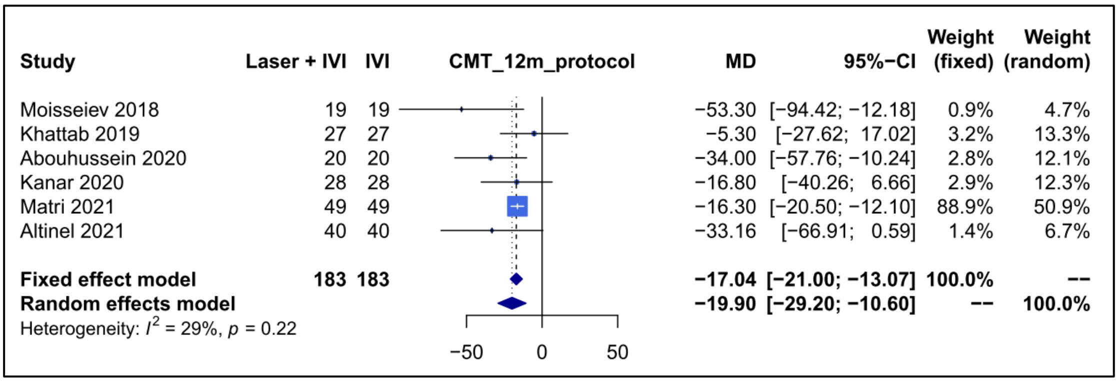

LogMarBCVA_3-month_exclude different protocol

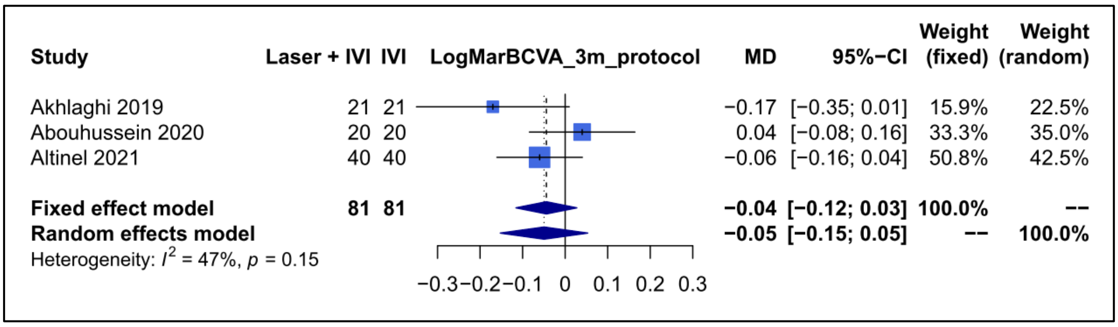

LogMarBCVA_6-month_exclude different protocol

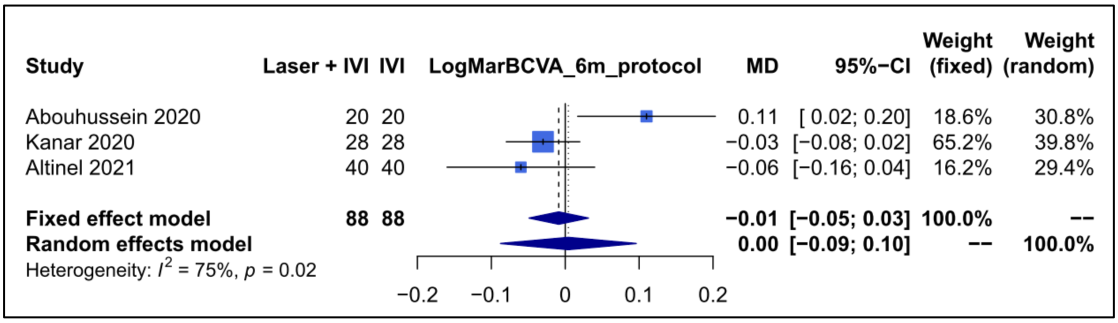

LogMarBCVA_12-month_exclude different protocol

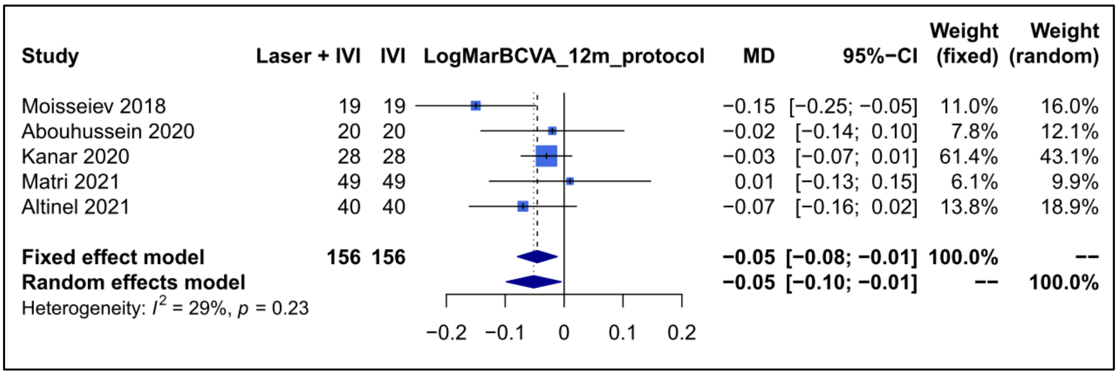

Total IVI Number_exclude different protocol

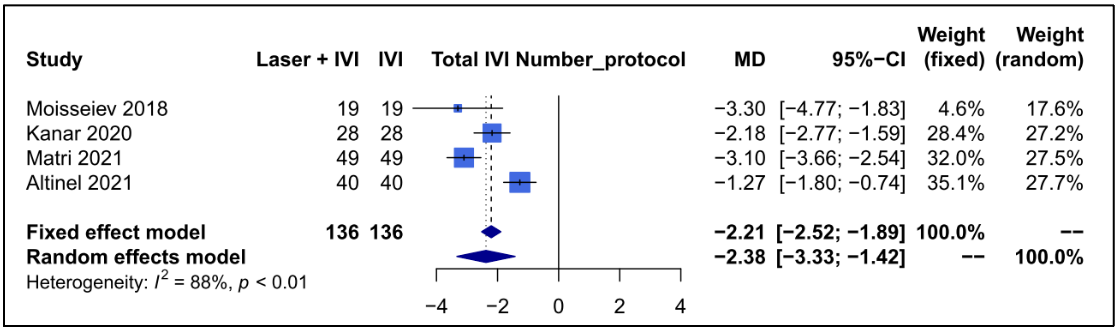

Figure S3. Subgroup Analysis of Outcomes for Different Anti-VEGF medications

CMT_3-month_ different anti-VEGF

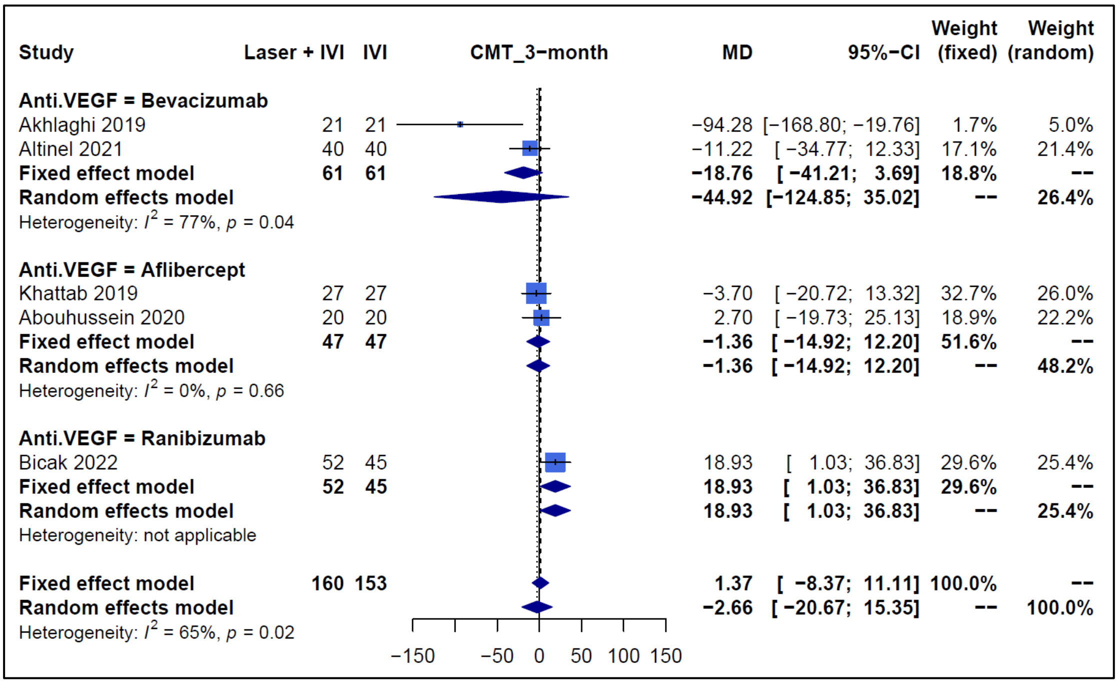

CMT_6-month_ different anti-VEGF

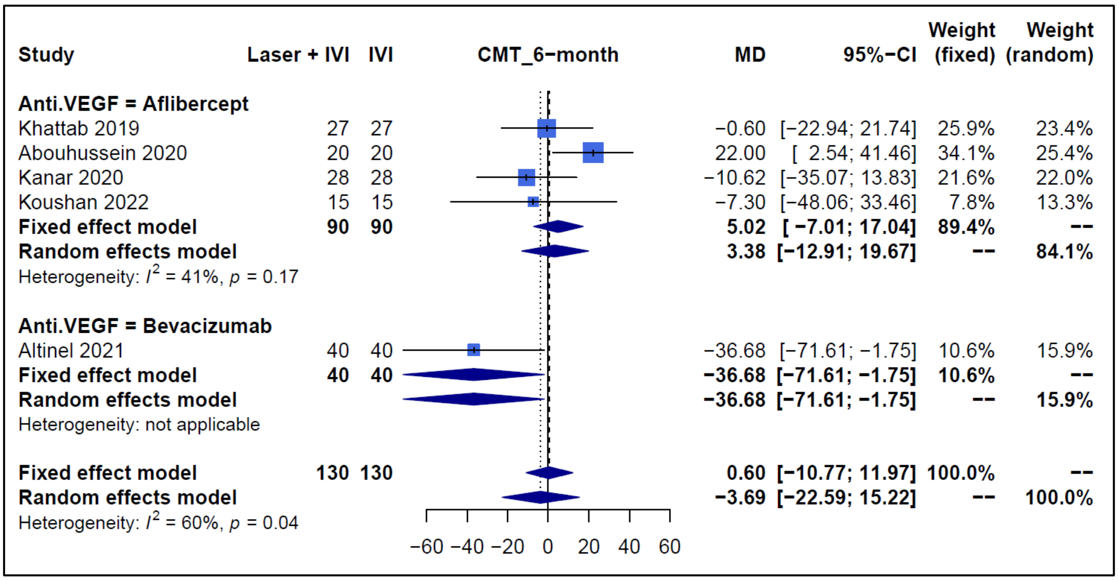

CMT_12-month_ different anti-VEGF

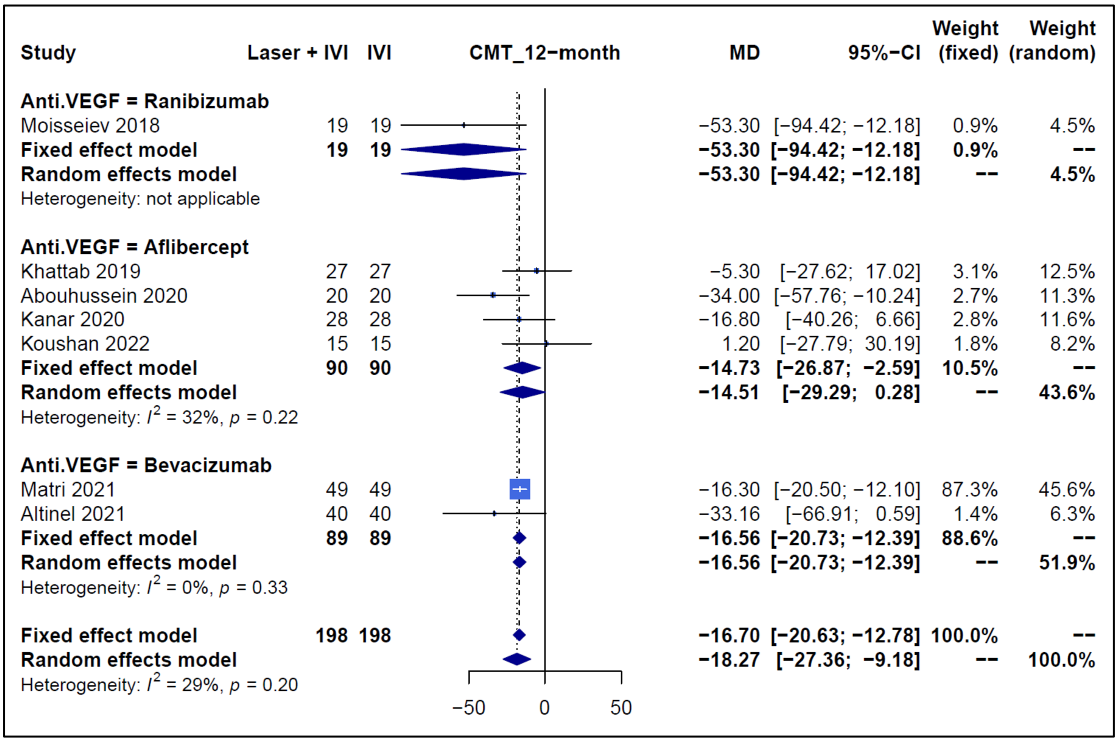

LogMarBCVA_3-month_ different anti-VEGF

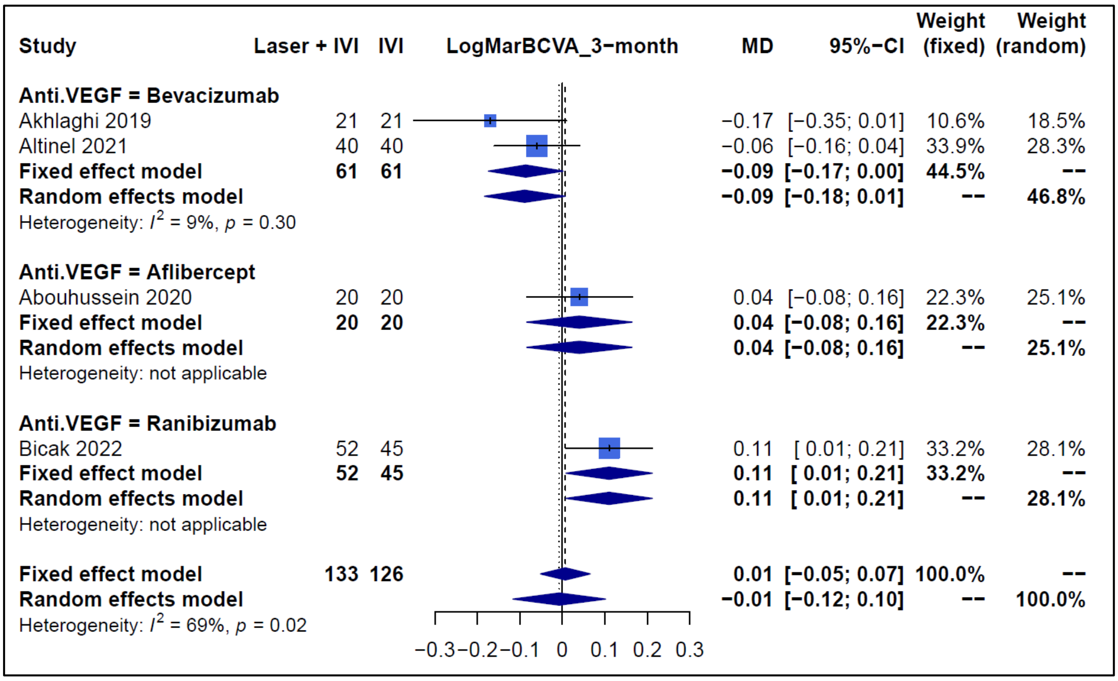

LogMarBCVA_6-month_ different anti-VEGF

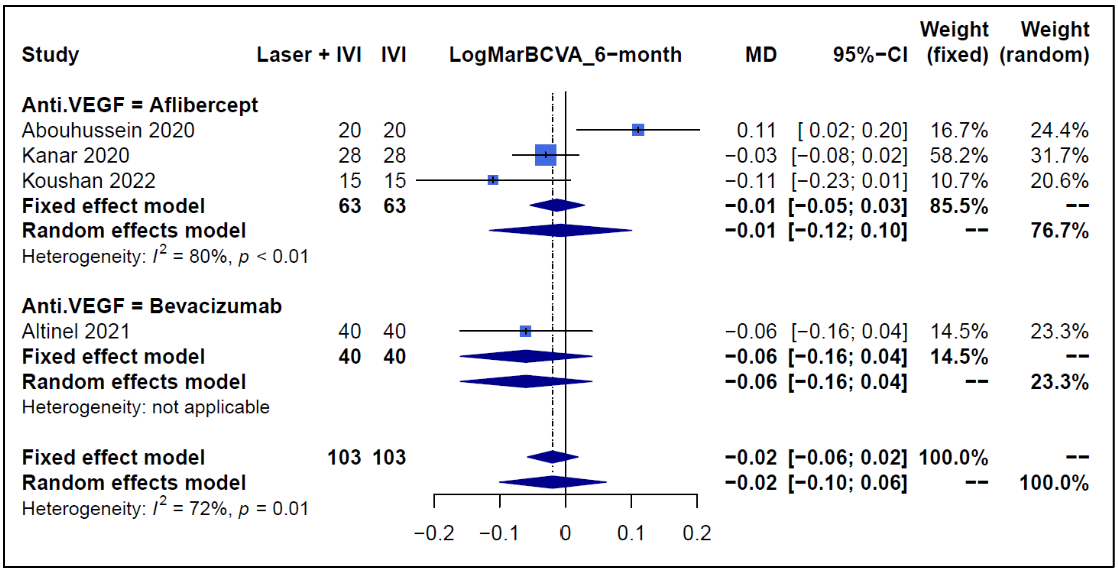

LogMarBCVA_12-month_ different anti-VEGF

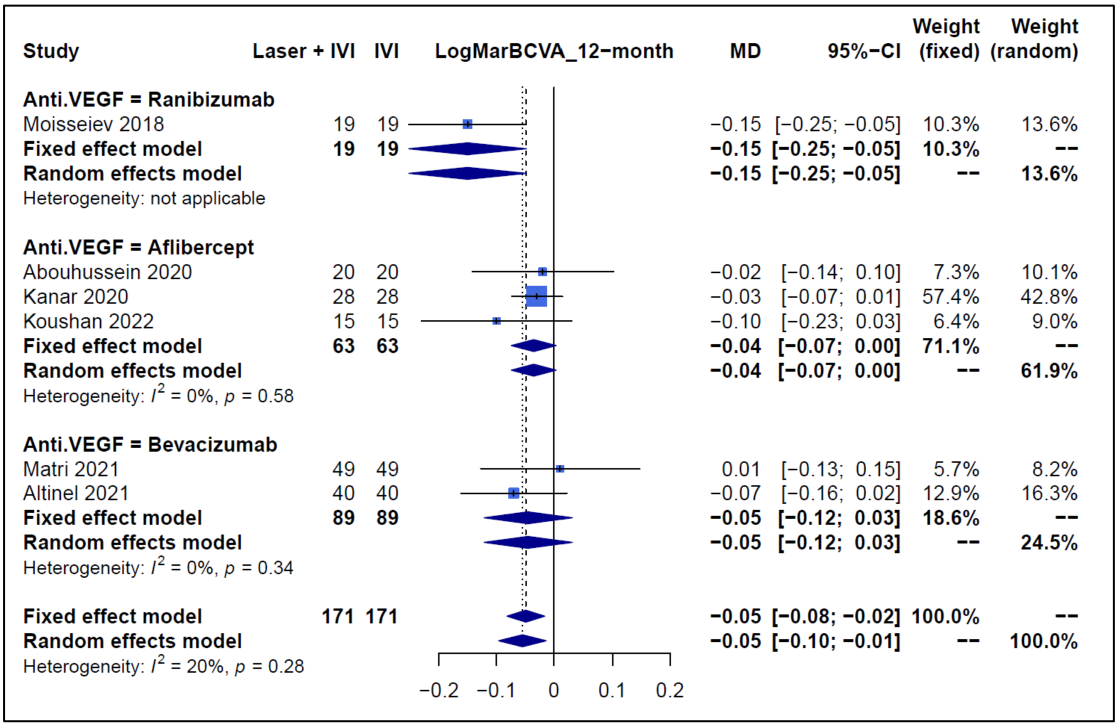

Total IVI Number_ different anti-VEGF

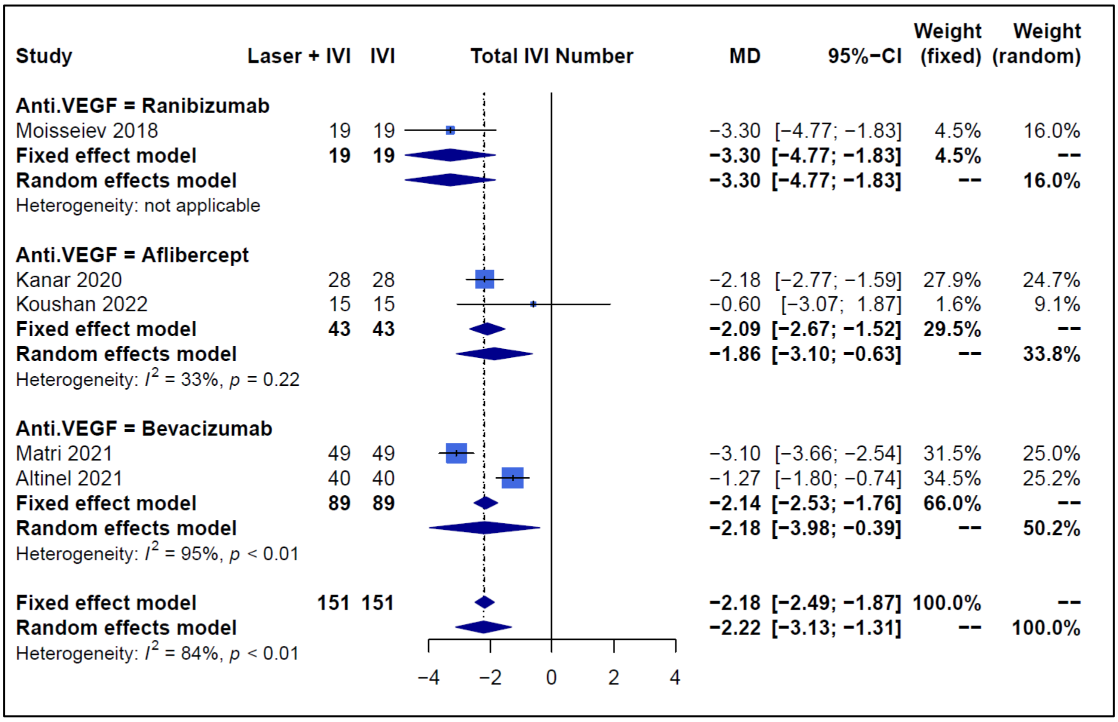

Supplement: Supplementary file 1 [file jcm-13-04782-s001.zip › jcm-3097549-supplementary.pdf]
